# Regulation of Cell Proliferation and Migration by Extracellular Phosphatidic Acid

**DOI:** 10.3390/biomedicines14030616

**Published:** 2026-03-10

**Authors:** Ana Gomez-Larrauri, Asier Benito-Vicente, Kepa B. Uribe, Cesar Martin, Antonio Gomez-Muñoz

**Affiliations:** 1 Department of Biochemistry and Molecular Biology, Faculty of Science and Technology, University of the Basque Country (UPV/EHU), P.O. Box 644, 48080 Bilbao, Bizkaia, Spain; ana.gomezlarrauri@osakidetza.eus (A.G.-L.); asier.benito@ehu.eus (A.B.-V.); kepa.belloso@ehu.eus (K.B.U.); cesar.martin@ehu.eus (C.M.); 2 Respiratory Department, Cruces University Hospital, 48903 Barakaldo, Bizkaia, Spain; 3 Department of Molecular Biophysics, Biofisika Institute, Consejo Superior de Investigaciones Científicas (CSIC), University of the Basque Country (UPV/EHU), 48940 Leioa, Bizkaia, Spain

**Keywords:** cell migration, cell proliferation, lysophosphatidic acid receptor, phosphatidic acid

## Abstract

Phosphatidic acid (PA) is increasingly recognized as an important endogenous regulator of cell proliferation and migration, playing relevant roles in physiology and pathology. However, the potential and prominence of extracellular PA in controlling cell functions are not so well established. The present review article has been undertaken to update and discuss the latest findings on extracellular PA as regulator of cell homeostasis, with special attention being paid to its role in the regulation of cell growth and migration. Specifically, exogenous PA potently stimulates myoblast proliferation and lung cancer cell migration, pointing to a critical role of this glycerophospholipid in the regulation of muscle cell regeneration and lung cancer dissemination. Interestingly, both of these actions are mediated through interaction of PA with lysophosphatidic acid (LPA) receptors and the subsequent activation of different signal transduction pathways. In particular, PA induces mitogen-activated protein kinase kinase (MEK)/extracellularly regulated kinases (ERK) 1 and 2, phosphatidylinositol 3-kinase (PI3K)/Akt, focal adhesion kinase (FAK)/Rac1, and Janus kinase-2 (JAK2)/signal transducer and activator of transcription 3 (STAT3). These findings may contribute to a better understanding of muscle cell biology and may help to develop new therapeutic strategies to treat lung cancer dissemination.

## 1. Introduction

Phosphatidic acid (PA) is a multifaceted bioactive glycerophospholipid that participates in the regulation of a variety of cellular functions, some of them being crucial for maintaining cell dynamics and homeostasis. Major roles of PA include its capacity to be the substrate of key enzymes to promote the synthesis of relevant glycerolipids, including structural membrane phospholipids, namely phosphatidylcholine (PC), phosphatidylethanolamine (PE), phosphatidylinositol (PI) and cardiolipin (CL) [1,2,3,4]. Moreover, PA is the precursor of energy-storing lipids, primarily triacylglycerol (TAG) [1], which is composed of a glycerol backbone and three fatty acyl chains, and is stored in lipid droplets in the adipose tissue. Another important feature of PA is that it can give rise to the formation of signaling molecules such as diacylglycerol (DAG), or lysophosphatidic acid (LPA) when it is acted upon by specific phosphatidate phosphohydrolase (PAP) activities or by A-type phospholipases, respectively [1]. Both DAG and LPA can regulate numerous cell functions. Of note, PA has been shown to participate in inflammatory responses. In particular, administration of PA to mice resulted in systemic inflammation through mechanisms implying the release of proinflammatory cytokines, such as tumor necrosis factor alpha (TNF-α), interleukin (IL) 6, or IL-1β, as well as nitric oxide (NO) and prostaglandin E2 [5]. In addition, PA has been shown to regulate autophagy [6]. In this regard, in a mouse model of major depressive disorder (MDD), injection of PA promoted autophagy in neurons, both in vitro and in vivo, through a mechanism involving regulation of ganglioside M1 levels, thereby correcting stress-induced alterations in the behavior of these mice [7]. Moreover, PA has been implicated in phagocytosis [8] and plays critical roles in membrane trafficking [9] and exocytosis events (reviewed in [10,11]). The present review particularly highlights the crucial roles of extracellular PA in the regulation of cell proliferation and migration with the purpose of delineating new avenues leading to developing novel therapeutic tools to treat cell growth- or migration-related illnesses.

## 2. Biosynthesis and Sources of PA

**Figure 1:**
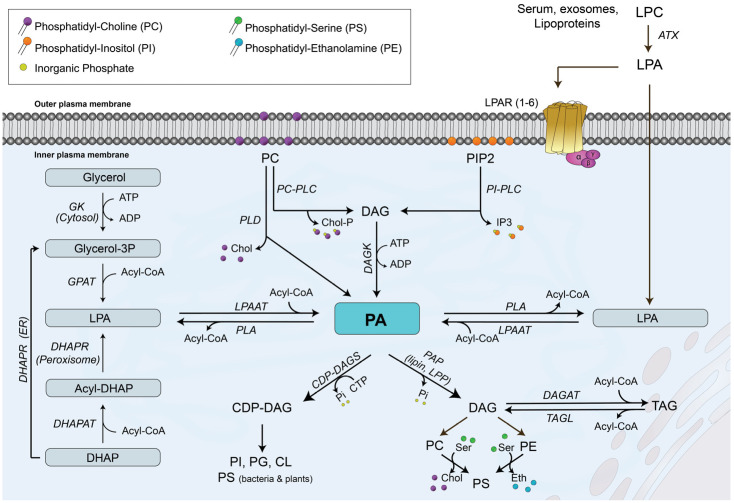
Biosynthetic pathways and metabolism of phosphatidic acid (PA). There are three major pathways leading to the biosynthesis of PA: (i) one pathway involves the degradation of phosphatidylcholine (PC) to diacylglycerol (DAG) by PC-dependent phospholipase C (PC-PLC) or phosphatidylinositol (PI)-dependent phospholipase C (PI-PLC) followed by phosphorylation of diacylglycerol (DAG) by DAG kinase (DAGK); (ii) a second pathway involves the degradation of PC directly to PA by phospholipase D (PLD) activity; (iii) the third pathway uses lysophosphatidic acid (LPA) to incorporate a fatty acyl chain into the molecule to generate PA directly in a reaction that is catalyzed by LPA acyl transferase (LPAAT). DHAP, dihydroxyacetone phosphate; DHAPAT, DHAP acyl transferase; DHAPR, DHAP reductase; GK, glycerol kinase; Glyc-3P, glycerol 3 phosphate. PA metabolism to PI, phosphatidylserine (PS), phosphatidylglycerol (PG), or cardiolipin (CL) involves the prior formation of cytidine diphosphate DAG (CDP-DAG) by CDP-DAG synthase (CDP-DAGS). Formation of PC, phosphatidylethanolamine (PE) or triacylglycerol (TAG) requires the degradation of PA to DAG by phosphatidate phosphohydrolase (PAP), an enzyme activity that includes lipin and lipid phosphate phosphatase (LPP) activities. ATX, autotaxin; Chol, choline; DAGAT, DAG acyl transferase; IP3, inositol trisphosphate; LPC, lysoPC; LPAR, LPA receptor; Chol-P, phosphocholine; PLA, phospholipase A; TAGL, TAG lipase. Further details are provided in Refs. [1,2,3,4,12,13,14,15].

PA is an intermediate metabolite that occurs at a branch point in the glycerolipid biosynthesis pathway. The first step in the de novo PA biosynthetic pathway is the formation of glycerol 3-phosphate (Glyc-3-P), which is the major acceptor of fatty acyl chains to form glycerolipids. There are two major pathways for generation of Glyc-3-P, depending on the type of tissue in which this molecule is generated. The existence of glycerol kinase (GK) in the liver allows the production of Glyc-3-P through direct phosphorylation of glycerol, whereas the source of Glyc-3-P in adipose tissue relies on glucose metabolism via the glycolytic pathway through generation of dihydroxyacetone phosphate (Figure 1). Once generated, Glyc-3-P can accept one fatty acyl chain to first form LPA, followed by the incorporation of a second fatty acyl chain to form PA in reactions that are catalyzed by Glyc-3-P acyl transferase (GPAT) and LPA acyl transferase (LPAAT), respectively [12]. There are four GPATs in mammalian cells, which are divided into two categories: mitochondrial and microsomal GPATs. GPAT1 and GPAT2 are mitochondrial enzymes found in the outer mitochondrial membrane, while GPAT3 and GPAT4 are microsomal GPATs located in the endoplasmic reticulum [12]. In addition, de novo synthesized PA is the main intermediate in the synthesis of TAG and glycerophospholipids. Specifically, degradation of PA by PAP-1 (also known as lipin) activity in the endoplasmic reticulum generates DAG, which is the precursor of PC, PE and TAG [1,13]. Alternatively, PA can be converted into cytidine diphosphate (CDP)-DAG by the action of CDP-DAG synthase in the present of CTP, and then converted to PI, phosphatidylglycerol (PG) or CL [1,13].

In addition to its biosynthesis in the endoplasmic reticulum, where PA can act as precursor of other phospholipids and TAG, there are three additional pathways that can generate PA at different locations within the cell, including the plasma membrane and the nucleus, where it can participate in cell signaling and/or gene expression events, respectively [14]. One pathway involves the acylation of LPA by LPA acyl transferase (LPAAT) at the plasma membrane [15]. In humans, there are six LPAAT isoforms, which can also localize to the Golgi and the endoplasmic reticulum [16]. A second pathway for biosynthesis of PA involves the phosphorylation of DAG by DAG kinase (DAGK) activity, which would generate PA with cell signaling properties. There are ten different DAGK isoforms, which can be localized to different compartments. Most DAGK isoforms are cytosolic, and some are associated with the endoplasmic reticulum, the Golgi, the nucleus, or the plasma membrane [17]. Of interest, some DAGKs, including DAGK-α, DAGK-ζ, DAGK-θ, and DAGK-δ1 can translocate from the cytosol to the plasma membrane in response to extracellular stimuli, and DAGK-ε, DAGK-κ, and DAGK-η2 are constitutively localized to the plasma membrane, where they can convert DAG into ‘signaling’ PA, a term adopted by Tanguy and coworkers to differentiate it from PA acting as a lipid precursor [11,17]. The third pathway for biosynthesis of the so-called ‘signaling’ PA involves the degradation of membrane phospholipids, namely PC, to produce PA and free choline by phospholipase D (PLD) activity at the plasma membrane or near the nucleus [11,14]. There are six different PLD isoforms in mammalian cells, which are named PLD1-6, with PLD6 also known as mitoPLD [14]. Only three PLD isoforms have well-established phospholipase activity to produce PA. In this regard, the most well-characterized PLD isoforms are PLD1, which is a 120 kDa protein that resides in the Golgi, lysosomes, and endosomes, and which can be recruited to the plasma membrane upon stimulation by exogenous stimuli, and PLD2, a 106 kDa protein that specifically localizes to the plasma membrane of cells [18]. Both PLD1 and PLD2 are involved in key cellular processes, namely cell proliferation, trafficking and migration [19], whereas PLD6 regulates mitochondrial dynamics and plays a role in Myc-mediated AMP-activated protein kinase activation and membrane fusion [18,20]. Of interest, PLD-derived PA has been shown to directly activate mTOR, a Ser/Thr kinase involved in cell proliferation, autophagy and migration [21,22].

Concerning extracellular PA, it can originate from several biochemical processes, or it can be present in different plasma components. In particular, cells can release PA-containing vesicles (such as exosomes or microvesicles) into the extracellular space during normal secretion or stress responses [23,24]. Also, PA is a key component of lipoproteins, which are present in plasma, but its primary role is different to that of the main lipids conforming the lipoprotein particles, namely TAG and cholesterol [25]. While the latter two lipids form the hydrophobic core of chylomicrons, very low density lipoproteins (VLDL), low density lipoproteins (LDL) and high density lipoproteins (HDL), PA is part of the hydrophilic shell of the lipoprotein particle [25] and might be able to interact with specific functionally specialized regions of the plasma membrane where particular proteins, receptors, channels, rafts, or molecular processes are concentrated. Also, PA is bound to serum albumin, the body’s main lipid carrier, for transport in the blood [26]. Albumin acts as a crucial vehicle, carrying this potent signaling molecule and other bioactive lipids to influence processes like cell growth and migration. Extracellular PA might also be generated from exogenous PLD. In fact, human plasma was shown to possess PLD activity [27], which may generate PA at the outer leaflet of the plasma membrane. Of interest, the concentration of PA found in human plasma has been reported to be in the range of 2.66–3.5 µM [28], or about 3.8 µM in healthy mice [7], but local concentrations produced upon PA release from cells might exceed those levels. It should also be taken into account that PA is enriched in extracellular vesicles, which can be secreted by most cell types, allowing PA to interact with the plasma membrane of cells [23,29,30]. In any case, extracellularly generated PA can regulate key biological activities, including cell proliferation and cell migration, as discussed below.

## 3. Regulation of Cell Proliferation by Extracellular PA

PA was previously demonstrated to exert growth factor-like actions [31]. Specifically, initial studies showed that PA induced the expression of the proto-oncogenes c-fos and c-myc and that it promoted DNA synthesis in Rat-1 fibroblasts [31,32]. The stimulation of DNA synthesis by PA was about 14.5 fold over the relative control value, and PA acted synergistically with insulin, which only stimulated DNA synthesis by 2.5 fold, to raise the value up to 31.7 fold [31]. Interestingly, the stimulation of DNA synthesis by PA was superior to that of LPA, which is also a potent stimulator of DNA synthesis and cell growth [31,32]. Also importantly, PA inhibited the activity of the GTPase activating protein (GAP) of Ras, a small homomeric G protein that is implicated in the regulation of normal cells, or transformed (cancer) cells when mutated [33]. The inhibition of GAP would maintain Ras in its activated form (bound to GTP) to continuously transmit mitogenic signals to the cells, an action that might lead to hyperplasia and tumor development.

**Figure 2:**
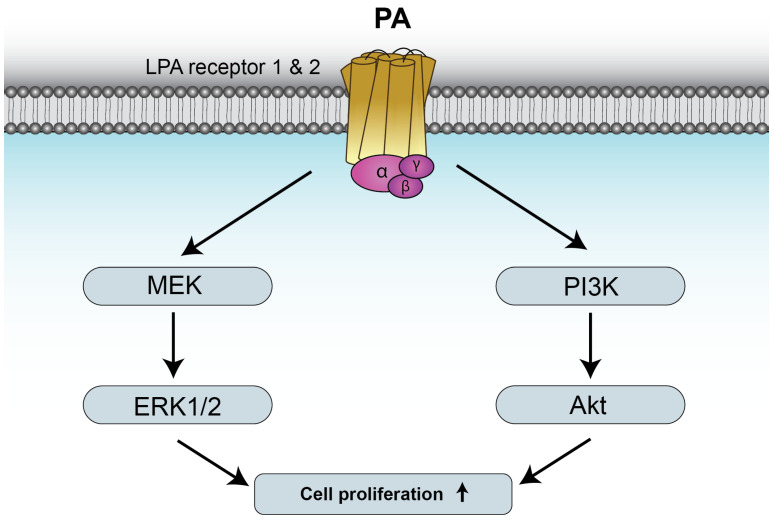
PA stimulates cell proliferation through binding to LPAR1 and 2. The mechanisms by which PA stimulates myoblast proliferation involve activation of the mitogen-activated protein kinase kinase (MEK)/extracellularly regulated kinases ERK1-2, and phosphatidylinositol 3-kinase (PI3K)/Akt pathways. Further details are provided in reference [41].

The mitogenic effects of PA have been mainly attributed to PA generated intracellularly, namely through stimulation of DAGK or PLD activities. In this connection, Fukami and Takenawa first reported that PA that accumulated in platelet-derived growth factor (PDFG)-stimulated Balb/c 3T3 mouse cells was a potent mitogenic signal for these cells [34]. Also, intracellularly generated PA, namely through PLD activation, can interact directly with mTOR to stimulate cell growth and metabolism [22] and to interact with caspase-8-induced cleavage of Bid to generate truncated Bid (tBid), which is a mediator of the lysosomal-mitochondrial pathway of apoptosis [35]. Moreover, intracellular PA plays an important role in the regulation of clathrin-mediated endocytosis of EGF receptors [36], and PLD2 is important for internalization of opioid and metabotropic glutamate receptors [18]. A highly relevant finding in the area of PA biology was that, in addition to generating PA, PLD2 also produced cyclic PA (1-acyl-2,3-cyclic glycerophosphate) [37]. Of note, a major action of cyclic PA is the inhibition of peroxisome-proliferator-activated receptor gamma (PPARγ), a transcription factor that drives adipocyte differentiation, lipid accumulation in macrophages, and arterial wall remodeling in rats in vivo [37]. The latter actions place intracellular PA as a relevant regulator of the activity of various receptors and the signaling pathways that they can trigger, by controlling their ability and distribution in cells. However, the occurrence of PA in extracellular compartments is compatible with the hypothesis that PA may interact with specific plasma membrane proteins or receptors to elicit some of its biological effects. In line with this hypothesis, Alderton et al. [38,39,40] suggested that PA was a G protein-coupled receptor agonist and showed that it upregulated the p42/p44 mitogen-activated protein kinase (MAPK) pathway in human embryonic kidney 293 cells. However, whether PA might be able to induce mitogenesis in these cells was not examined. Of note, it was recently reported that extracellular PA stimulated DNA synthesis and cell division in mouse myoblasts through a mechanism involving activation of the mitogen-activated protein kinase kinase (MEK)/extracellularly regulated kinase (ERK) 1-2 (p44/p42 MAPK) and phosphatidylinositol 3-kinase (PI3K)/Akt pathways (Figure 2). However, the MAPKs p38 and c-jun N-terminal kinase (JNK) were not involved in this process [41].

The mitogenic effects of PA could be reproduced by using exogenous bacterial PLD, which generates PA at the plasma membrane of cells. Of note, the stimulation of cell proliferation by PA was potently inhibited by pretreatment of the myoblasts with pertussis toxin (Ptx), which specifically inhibits heterotrimeric GTP binding proteins of the Gi family, suggesting the implication of a G protein-coupled receptor (GPCR) in this process. Ptx also blocked PA-mediated phosphorylation of Akt and ERK1-2, which are relevant regulatory kinases of mitogenesis and cell survival [41]. The latter observations led to the hypothesis that extracellular PA might exert its mitogenic effects through interaction with a plasma membrane (G(i)PCR). However, although the existence of a specific receptor for PA is a matter of current investigation, and efforts to find such receptor have so far rendered unsuccessful results, it was observed that blockade of LPAR 1 and 2 (using either specific siRNA to silence the genes encoding these receptors, or pharmacological inhibitors), completely abolished PA-stimulated Akt and ERK1-2 phosphorylation and cell proliferation. These observations are consistent with those of Fujiwara et al. [42] showing that LPAR could be activated by PA regioisomers and analogs, albeit with different potency. Also, although some initial studies argued against the ability of PA to elicit biological actions in its own right and attributed its effects to PA-derived LPA, studies using radioactive [^14^C]PA demonstrated that PA-stimulated myoblast proliferation did not rely on its conversion into LPA [41]. Moreover, PA was able to completely displace LPA that was tightly bound to its cell membrane receptors, whereas sphingosine 1-phosphate (S1P), which is structurally similar to LPA, or ceramide 1-phosphate (C1P), which is structurally related to PA, did not alter LPA binding to cell membranes [41]. Taken together, the latter results indicate that PA might stimulate myoblast proliferation through a mechanism involving its interaction with LPAR 1 and 2 and subsequent stimulation of the PI3K/Akt and MEK/ERK1-2 signaling pathways [41]. Apart from being involved in PA-stimulated cell proliferation, LPAR2 agonists have been reported to provide new therapeutic options for secretory diarrhea and gastric erosions caused by nonsteroidal anti-inflammatory drugs [43]. However, PA is not a universal mitogen, as it does not promote proliferation of human lung cancer cells (N. Presa et al. unpublished work), and this may be the case for other cell types, thereby indicating that the effects of exogenous PA may be cell-type specific. It should be noted that the mitogenic properties of PA may have been well conserved through evolution, as PA was also able to stimulate cell cycle progression and proliferation in muscle cells of the fish turbot (*Scophthalmus maximus*) [44].

## 4. Regulation of Cell Migration by Extracellular PA

**Figure 3:**
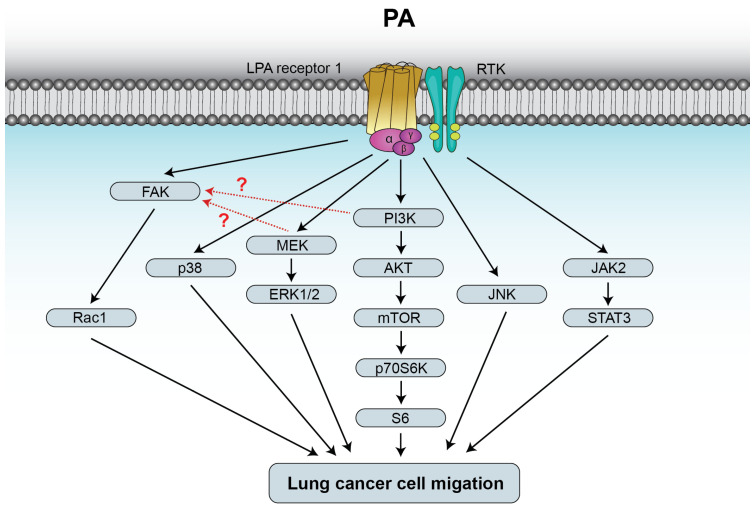
PA stimulates lung cancer cell migration through binding to LPAR1. The mechanisms by which PA stimulates lung cancer cell migration involve activation of the mitogen-activated protein kinase kinase (MEK)/extracellularly regulated kinase ERK1-2, phosphatidylinositol 3-kinase (PI3K)/Akt/mammalian target of rapamycin (mTOR)/p70S6K/S6, and focal adhesion kinase (FAK)/Rac1 pathways. In A549 human lung cancer cells, activation of FAK by PA seems to be downstream of MEK and PI3K (red dotted lines). PA-stimulated cell migration also includes activation of p38 or c-jun N-terminal kinase (JNK). In addition, binding of PA to LPAR1 leads to transactivation of a receptor tyrosine kinase (RTK) and subsequent stimulation of the Janus kinase 2 (JAK2)/signal transducer and activator of the transcription 3 (STAT3) pathway. Further details are provided in references [21,58].

Cell migration is crucial for regulation of physiologic processes including embryonic development, immune responses, wound healing, and homeostasis in general, with errors linked to various pathologies, including chronic inflammatory diseases and cancer cell metastasis [45,46,47]. A myriad of external signals can induce directed cell migration, many of which are chemical cues including bacterial peptides, growth factors and chemokines [48]. Of note, some bioactive lipids, including glycerophospholipids such as LPA and PA, or sphingophospholipids such as S1P or C1P, have also been shown to regulate cell migration [49,50,51]. Concerning PA, it was shown that exogenous PA acts as a leukocyte chemoattractant, as membrane-soluble dioleoyl-PA induced actin polymerization and chemotaxis of human neutrophils and HL-60 human leukemia cells [52]. Although more than 40 different species of PA can be found in different cell types, raising the possibility that they may regulate different cell functions [53], dipalmitoyl-PA and 1-palmitoyl, 2-oleoyl-PA were equally potent at stimulating lung cancer cell migration [21]. Concerning cell migration, it was shown that PA stimulation of human neutrophil migration involved activation of the mTOR/p70S6 kinase pathway, but the chemotactic effect of PA could only be inhibited by about 30% with specific mTOR siRNA, indicating the existence of an mTOR-independent mechanism for p70S6K activation [52]. In this connection, it should be noted that exogenous PA was shown to trigger the formation of intracellular PA through stimulation of PLD activity in rat fibroblasts [32] and corneal epithelial and Madin Darby canine kidney cells [54], and that similar outcomes were observed when human neutrophils were challenged with exogenous PA [52]. Hence, full activation of mTOR may require the intervention of both extracellular PA and PLD-derived intracellular PA. Interestingly, overexpression of PLD2, and, to a lesser extent, PLD1, led to upregulation of S6K activity, whereas PLD silencing decreased p70S6K activation, suggesting that both extra and intracellular PA were needed to fully stimulate the mTOR/p70S6K pathway [52]. More recently, exogenous PA was shown to stimulate human epithelial lung cancer cell migration through rapid stimulation of the MAPKs ERK1-2, p38 and JNK [21]. Of note, Ptx completely blocked phosphorylation of the latter MAPKs and cell migration, suggesting the intervention of a G(i)PCR in the chemotactic effect of PA. Nonetheless, in macrophages, exogenous PA reduced C1P-stimulated migration through binding to the putative C1P receptor [55]. Although, as mentioned earlier, exogenously applied PA can enter the cells, specific blockade of LPAR1 completely abolished both PA-stimulated MAPK phosphorylation and lung cancer cell migration, suggesting that PA may exert its chemotactic effects through binding to this receptor [21]. It should be noted that, although different molecular species of PA with long fatty acyl chains (dipalmitoyl-PA or 1-palmitoyl, 2-oleoyl-PA) stimulated cell migration to a similar extent, Fischer et al. [56] showed that short-chain PA subspecies are subtype-selective antagonists of LPAR. In addition to stimulating MAPK phosphorylation (activation), PA also activated the JAK2/STAT3 pathway, and either inhibition of JAK2 or STAT3 blocked PA-stimulated lung cancer cell migration, suggesting that this pathway may also be relevant in the regulation of cell migration by PA [21]. The JAK2/STAT3 pathway is usually activated through binding of growth factors or chemokines to receptor tyrosine kinases (RTK), but binding of PA to this type of receptors has not as yet been reported. Nonetheless, it is likely that PA can lead to activation of RTK by binding to a GPCR through a process called transactivation. In fact, activation of some RTK has been demonstrated after the binding of LPA to one of its six GPCRs [57]. In a more recent study, PA was also shown to stimulate the PI3K/Akt pathway in lung cancer cells though binding to LPAR1. The latter action also led to activation of mTOR, which is a direct target of Akt, and to downstream phosphorylation of p70S6K, followed by S6 protein activation [58]. The latter study also suggested participation of the focal adhesion kinase (FAK)/Rac1 pathway in this process. Although FAK has been reported to be upstream of ERK1-2 and PI3K activation [59,60], this is not clear for PA stimulation of ERK1-2 in lung cancer cells, as the latter kinases are phosphorylated some time before (5–10 min) that FAK activation (20 min) (Figure 3). It was proposed that the pathological relevance of lung cancer cell migration stimulation through binding of PA to LPAR1 relies on the fact that PA can substitute for LPA when generation of the lysophospholipid is altered or impaired, so as to facilitate LPAR1-mediated cell migration [21].

## 5. Other Biological Actions of Exogenous PA

Another relevant action of PA is the regulation of membrane or vesicular trafficking. Although this PA action has been mainly attributed to intracellularly generated PA (PLD-derived) [22], regulation of the insulin-dependent glucose transporter GLUT4 trafficking and insulin secretion from pancreatic β-cells was also ascribed to exogenous PA [22]. Also, it has been shown that physiologic concentrations of exogenous PA regulate autophagy in mouse neurons both in vitro and in vivo. In particular, the inhibition of autophagy elicited by corticosterone, which reduces the levels of ganglioside M1, was normalized upon injection of the mice with PA [7]. Also important is the fact that exogenous PA reduces acetaminophen-induced liver injury [61] and that it stimulates hair growth [62]. In particular, it was shown that, contrary to LPA, exogenous PA possesses intensive growth promotional effects on hair epithelial cells and keratinocytes, both in vitro and in vivo through a mechanism involving activation of extracellular MEK1-2 [62]. Although these actions of PA are beneficial for cells, exogenous PA can also be detrimental as mentioned in this review and because it can induce thrombogenic activities in erythrocytes. In particular, the mechanism whereby PA can lead to the development of cardiovascular disease involves exposure of phosphatidylserine (PS) in erythrocytes, an action that increases blood clotting, ultimately contributing to thrombosis [63]. Exogenous PA was also observed to produce a concentration-dependent increase in intracellular free calcium levels in cultured vascular smooth muscle cells [64], and it played a relevant role in the coupling of the ERK cascade through the formation of PA-enriched membrane microdomains [65]. Also of interest, in non-mammalian vertebrates such as broiler chickens, inclusion of PA in their diet improved body weight and production efficiency [66]. Finally, it should be pointed out that PA is critical for the recruitment of Raf-1 to the plasma membrane [67] and was shown to overcome fetal growth deficits and maternal uterine artery dysfunction in a model of fetal alcohol syndrome, which develops when a pregnant mother consumes alcohol [68]. Hence, the effects of extracellular PA are multiple and relevant in the regulation of cell biology.

## 6. Conclusions

The occurrence of PA in the extracellular milieu led to the hypothesis that it can elicit some of its biological effects through interaction with specific functionally specialized regions of the plasma membrane, including receptors or receptor-associated proteins. Whilst endogenous PA can bind and activate various intracellular enzymes or effectors to regulate many biologic cell functions, the biological actions of exogenous PA remain poorly characterized. PA is a bioactive phospholipid that is localized to intracellular compartments as well as to extracellular structures. Specifically, PA can be found in extracellular vesicles (exosomes), lipoproteins, or serum albumin and can also be generated by exogenous PLD (i.e., from bacteria), which can degrade PC that is located in the outer leaflet of the plasma membrane to form PA. Although the extracellular concentration of PA has been reported to be around 3.5 µM, it is possible that higher concentrations might occur locally. Hence, in addition to binding to cell membrane receptors, at elevated levels, PA may more readily integrate into intracellular compartments, eventually accumulating to concentrations sufficient to initiate specific metabolic responses.

In the present review, we report on the major roles of extracellular PA, namely regulation of cell proliferation and migration, and highlight the mechanisms by which PA exerts these actions. A major finding in this regard was that exogenous PA utilizes LPA receptors to elicit at least some of its biological activities. Specifically, PA promotes cell proliferation through binding to LPAR1 and 2 and regulates lung cancer cell migration through binding to LPAR1. In either case, binding of PA to the LPA receptors triggered activation of the MEK/ERK1-2 and PI3K/Akt pathways, with p38, JNK and FAK/Rac1 being also relevant for regulation of lung cancer cell migration. In some cases, binding of PA to LPAR may cause transactivation of RTK receptors, leading to stimulation of additional signaling pathways, including the JAK/STAT pathway, and/or amplify other LPAR-triggered pathways. A further important outcome of PA interacting with LPAR is that PA can take the place of LPA when LPA levels are low, enabling LPAR activation even when LPA is not present at sufficient concentrations. Since LPAR signaling is highly pathophysiologically relevant, small changes in receptor activation may significantly alter disease pathways, a matter that should be taken into consideration when developing LPAR-based therapies.

It should finally be pointed out that novel analytical strategies such as new imaging techniques to improve spatiotemporal localization of PA, mass spectrometry of high resolution, and photoswitchable analogues of PA [53] will be paramount to monitor PA distribution and metabolism, so as to understand the role played by this unique glycerophospholipid in cell biology.

## Data Availability

No new data were created or analyzed in this study. Data sharing is not applicable to this article.

## Abbreviations

C1P	ceramide 1-phosphate
CL	Cardiolipin
DAG	Diacylglycerol
DAGK	diacylglycerol kinase
ERK	extracellularly regulated kinase
FAK	focal adhesion kinase
Glyc-3-P	glycerol 3-phosphate
GPAT	Glyc-3-P acyltransferase
GPCR	G protein-coupled receptor
IL	Interleukin
JAK	Janus kinase
JNK	c-Jun N-terminal kinase
LPA	lysophosphatidic acid
LPAAT	LPA acyl transferase
LPAR	LPA receptor
LPP	lipid phosphate phosphatase
MAPK	mitogen-activated protein kinase
MEK	mitogen-activated protein kinase kinase
NO	nitric oxide
mTOR	mechanistic (mammalian) target of rapamycin
PA	phosphatidic acid
PC	phosphatidylcholine
PE	phosphatidylehanolamine
PI	phosphatidylinositol
PI3K	phosphatidylinositol 3-kinase
PLD	phospholipase D
STAT	signal transducer and activator of transcription
S1P	sphingosine 1-phosphate
TAG	triacylglycerol
TNF	tumor necrosis factor
